# Interactive Association of Negative Creative Thinking and Malevolent Creative Thinking

**DOI:** 10.3389/fpsyg.2022.939672

**Published:** 2022-07-13

**Authors:** Xinyu Dou, Xinyan Dou, Lin Jia

**Affiliations:** ^1^School of Music, Zhengzhou University, Zhengzhou, China; ^2^School of Information Engineering, Shandong Vocational and Technical University of International Studies, Rizhao, China

**Keywords:** negative creative thinking, malevolent creative thinking, linkage model, value-add, linkage

## Abstract

With the existing research available on negative and malevolent creativity, this paper proposes a more narrowly defined concept: the bi-directional relationship between negative and malevolent creative thinking, which is intended to clarify the way forward for research in the area of negative and malevolent creativity. This paper uses qualitative research to identify and correlate an individual's concept of negative and malevolent creativity and uses a Inductive reasoning methodology to outline a preliminary theory. Following this, the preliminary theory was returned to the primary sources for validation, continuous optimization, and presentation. To better demonstrate the bidirectional linkage mechanism of thinking between the two types, this paper constructed a model to describe the relationships between the variables. This study concludes that negative creative thinking is a kind of native thinking based on personal interests that are developed to emphasize the benefits of an individual's interests, while malevolent creative thinking is a kind of native thinking based on the value-added of personal interests and is deliberately harmful. Both types of thinking share a value orientation, environmental stimulation, and subjective motivation. However, they differ in terms of value goals, ways of thinking, and the scale of the subject. It is concluded from the intrinsic thinking logic of individual thinking as well as the temporal dimension of the individual creative process that value-added and intentions to exploit others are factors that drive the transformation of negative creative thinking into malevolent creative thinking, and personal intention is a vital factor for establishing a linkage between negative and malevolent creative thinking.

## Introduction

In the field of education there is a consensus almost unanimously that creativity can be taught (Cropley, 1992; Runco and Chand, 1995; Amabile et al., 1996; Wilson, 2005; Baer and Kaufman, 2006; Kaufman and Beghetto, 2009). Creativity education consists of two main aspects. The first aspect is about teaching and learning, that is, how to provide creative and innovative educational practices that stimulate higher-level thinking and opportunities to explore multiple alternative solutions (Cropley, 1992; Fryer, 1996; Lin, 2011). The second aspect is the creation of a supportive environment, which can stimulate learners' motivation and creative behavior (Torrance, 1995; Collins and Amabile, 1999; Hennessey, 2007; Lin, 2011). In creativity education, it is largely the teacher who guides students through the creative process.

Scholarly research on creativity has mostly focused on the forms of creation and its originality and appropriateness (Runco and Jaeger, 2012). In other words, it is the study of the positive side of creativity (Cropley et al., 2014). However, creativity does not always lead to positive outcomes (Clark and James, 1999; Cropley and Cropley, 2011; Harris and Reiter-Palmon, 2015; Ligon et al., 2017), and Carl (1954) illustrates the existence of another side of creativity. Identifying the connotations of negative creative thinking and malevolent creative thinking as well as clarifying the differences and connections between the two types of thinking will help rationalize the relevant academic discourse system and standardize our research; at the same time, it will also help researchers to think more accurately about the thinking cultivation paths in the creative process and thus further promote the development of creative education. Therefore, this paper will separately discuss the core connotations of negative creative thinking and malevolent creative thinking, identify the similarities and differences in connotations between them, and propose a linkage model of negative creative thinking and malevolent creative thinking to remove conceptual barriers for the development of thinking research and provide a foundation for further research on the interaction mechanism between negative creative thinking and malevolent creative thinking.

## Methodology

Through qualitative research, this paper aims to inductively advance negative, malevolent creativity. This study was conducted using the Inductive reasoning method. Initially, a multistage data collection process was conducted to distill key information to be able to introduce new models from known theories (Strauss and Corbin, 1990, 1998; Polkowski, 2011). In the next step, a comparative analysis of the information that continues to emerge utilizes the concepts of negative and malevolent creativity, compares these concepts, considers the differences and similarities that exist between them, and ties these differences and similarities together. The preliminary hypothesis is: that there is an interactive association between the two types of thinking. It is then returned to the original source for validation and the existing theory is continuously optimized to make it more precise. Finally, the theory is explained, layer by layer, by describing the relationship between the two thinkings, as an answer to the research question. For better display of the linkage relationship, it is proposed to be presented in the form of visual images.

## Concepts Definition

### The Connotation of Malevolent Creative Thinking

Malevolent creative thinking is the creation of products that cause harm to humans and society and is therefore defined as creativity that intentionally leads to harmful or unethical outcomes (Ning and Jing, 2016). Malevolent creative thinking is not only associated with terrorism and crime (Cropley et al., 2008; Gill et al., 2013), but ordinary people also generate malevolent creative thinking. That is caused by a number of situational factors (Barbot et al., 2020). For example, dissatisfaction with society (Clark and James, 1999; Harris and Reiter-Palmon, 2015; Gutworth et al., 2016; Kapoor and Khan, 2019). Second, low emotional intelligence (EI) can also produce malevolent creative thinking (Harris et al., 2013). In addition, negative personality traits can also contribute to malevolent creative thinking, for example, trait physical aggression (Lee and Dow, 2011), implicit aggression, low pre-mediation (Harris and Reiter-Palmon, 2015), and low emotional intelligence (Harris et al., 2013). Malevolent creative thinking is characterized by intentional harm (Cropley et al., 2008).

### The Connotations of Negative Creative Thinking

Negative creative thinking is associated with narcissism, mental illness, and Machiavellianism (Hansika, 2015). Negative creative thinking is an intersection of originality and value as well, and is the use of creative processes to achieve negative goals, but without the intention of intentional harm (Clark and James, 1999; James et al., 1999; Kapoor, 2018). This behavior prioritizes self-service.

### Identifying the Connotation of Negative Creative Thinking and Malevolent Creative Thinking

#### Common Ground

There are high similarities between the two in the following 3 areas: First, the value orientation converges (Value). Both Malevolent Creative Thinking and Negative Creative Thinking are at the highest level of higher-order thinking and are usually closely linked to design thinking, analytical thinking, and critical thinking to achieve personal goals through the act of problem-solving. The intersection of originality and value is the value orientation of both malevolent creative thinking and negative creative thinking. The second is environmental stimulation (Environment). Kapoor and Khan (2019) provide an explanation for the situational variable that individuals are more willing to think malicious or negative creative thoughts in response to negative or unjust situations (Clark and James, 1999; Harris and Reiter-Palmon, 2015; Gutworth et al., 2016; Kapoor and Khan, 2019). The third is subjective motivation (Motivation). Both types of thinking are conscious and purposeful, and both require the support of personal intention factors (Mueller et al., 2012; Gutworth et al., 2016). When an individual's creative potential and tendency to react maliciously are controlled, the probability of malicious or negative thoughts being generated is reduced (Xu et al., 2021, 2022; Wang et al., 2022).

#### Connotation Differences

##### Different Value Goals

There is a threshold between malevolent creative thinking and negative creative thinking, which is the balance between individual and social interests (Mueller et al., 2012; Gutworth et al., 2016). Malevolent creative thinking intentionally causes harm to others and society in order to maximize personal interests (Eisenberger and Shanock, 2003; Cerasoli et al., 2014; Bochkova and Meshkova, 2019). While negative creative thinking seeks to achieve negative self-centered goals by creating new pathways that do not harm others and do not involve intentional destruction of the public good. While personal interest is the core of both types of thinking, whether or not one intends to harm others is the key to defining them (Cropley et al., 2008; Ning and Jing, 2016). The transformation process from negative creative thinking to malevolent creative thinking involves many elements, such as risk-taking.

##### Thinking Styles

Negative creative thinking is the generation of negative ideas and the development of ideas to achieve personal negative ideas and purposes (Clark and James, 1999; James et al., 1999; Kapoor, 2018); malevolent creative thinking is the creative approach to achieve maximum value-added for self-interest and is deliberately harmful in nature. Malevolence covers the highest level of negative expression. Negative creativity is the satisfaction of an individual's expected benefits, and its thought process includes solving problems as well as achieving some practical utility (James et al., 1999; Kapoor, 2018). In contrast, malevolent creation is the infinite amplification of value in spite of everything and based on negative creative thinking, it includes the idea of intentionally harming others to satisfy one's own malicious psychological achievements (Cropley et al., 2008; Runco, 2010; Ning and Jing, 2016). It can be seen that negative creative thinking is the original conception to achieve the personal benefit, and malevolent creative thinking is the original conception to add value and satisfy evil psychological fulfillment. Generally speaking, malice begins when higher added value emerges and has a motivation for change.

##### Different Subject Sizes

The process of negative creation is mostly based on a single creative subject; while the process of malicious creation is logically individual-based, but actually focuses on other aspects because of its nature of intentional harm (Hunter et al., 2022). Malevolent creation and negative creation are both systematic creations, relying on individuals to complete the process of transformation from creation to value. However, the process of value calculation and evaluation is more important in the development of malevolent creative thinking than negative creative thinking and contains more factors (Hunter et al., 2022). For example, making explicit and structuring unclear values as well as psychological satisfaction from intentional harm to the individual. As a result, malevolent creative thinking requires more consideration of collaboration with other factors and a larger scale of thinking subjects.

The preceding concepts of negative and malevolent creativity revealed considerable disparities in the conceptual definitions of negative and malevolent creative thinking. Negative creative thinking is a sort of original thinking that is founded on personal interests and then develops to emphasize the benefits of individual self-interest; malevolent creative thinking is original thinking that is based on the value-added of personal interests and has the nature of purposeful harm. In terms of value orientation, contextual stimulation, and subjective motivation, the two styles of thinking overlap. They differ, however, in terms of value goals, thinking styles, and subject size. We propose a more narrowly defined concept: the bi-directional linkage mechanism of negative-malevolent creative thinking, based on the intrinsic thinking logic of individual thinking and the temporal dimension of the individual creative process, which will contribute to the growth of meaningful research.

## Thinking Linkage

Gutworth et al. (2016) argued that the nature of the goal and the instructions for satisfying stated goals explain whether individuals produce malevolent creativity. Malevolent creative thinking is the creation of products that cause harm to humans and society and is therefore defined as creativity that intentionally leads to harmful or unethical outcomes (Ning and Jing, 2016). Negative creative thinking is the use of creative processes to achieve negative goals but without the intention of intentional harm (Clark and James, 1999; James et al., 1999; Kapoor, 2018). The two are closely related to the process of thought generation. From the above-mentioned common ground and connotational differences, negative creative thinking and malevolent creative thinking have an important linkage, namely value-added. Value is a linkage node in the transformation of negative creative thinking to malevolent creative thinking. Value is, on the one hand, the outcome of creative thinking generation and, on the other hand, the base point for negative and malevolent assessment. Therefore, personal intention toward value is a key stage in the linkage of negative creative thinking to malevolent creative thinking. Before this stage, negative creative thinking is dominant and ideas are highly individualized and self-serving, while after this stage, thinking needs to include risk-averse elements and make choices after considering multiple factors to form the final creative outcome (Mumford and Hunter, 2005; Mueller et al., 2012; Blank, 2013). The path from negative creative thinking to malevolent thinking is not unidirectional. In the process of thinking transformation, value is used as the linkage node between the two types of thinking, and personal intention is used as the determining factor leading to the mutual transformation between negative creative thinking and malevolent creative thinking.

**Figure 1:**
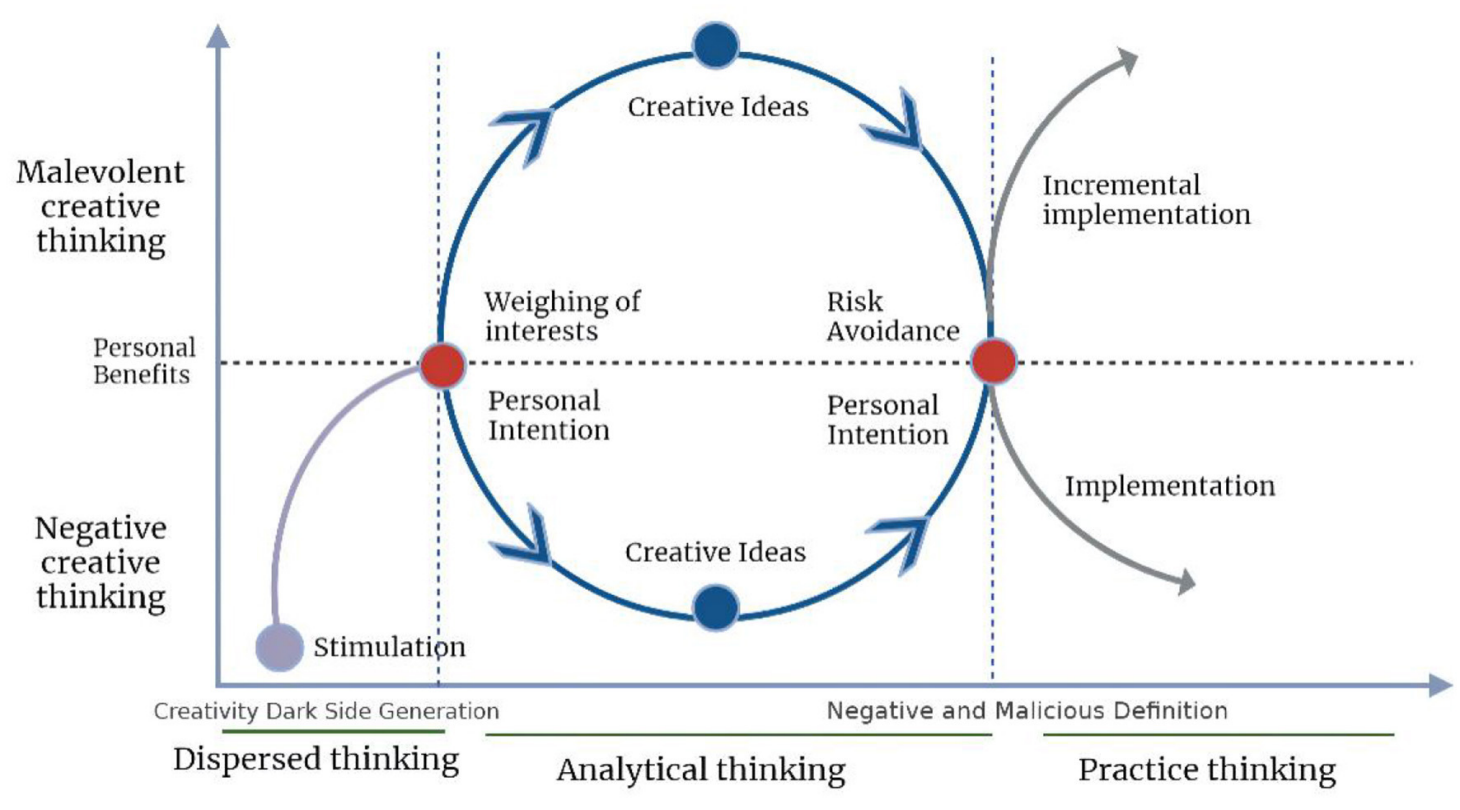
Negative-malevolent thinking interconnection model (NMTIM).

Creation is an evolving thought process (Zehui et al., 2019). Logic deduced the relationship between the variables of negative-malicious creative thought based on the temporal dimension of the creative process and the findings of previous investigations. We built a model of linkage between individual negative creative thought and malevolent creative thinking to better show the linkage relationship (Figure 1). In the chronological dimension of individual development, the interconnection model of negative and malevolent thinking is not abrupt and discontinuous, but rather a continuous spectrum with different emphases. In the initial stage of creative stimulation, the environment stimulates creative drive (Hennessey, 2007; Lin, 2011). Changes in situational variables, such as unjust environments (Harris and Reiter-Palmon, 2015; Gutworth et al., 2016; Kapoor and Khan, 2019), and environments with violent elements (Malik et al., 2020), can cause individuals' creative motivation to shift, resulting in the emergence of negative creative thinking (De Jesus et al., 2013). Malevolent thinking is further stimulated by calculations and reevaluations of personal interests, or by failed responses to creative stimulation events and conflict resolution. As a result, negative creative thinking is gradually transformed into malicious creative thinking. Of course, not all links between negative and malevolent creative thought develop gradually from negative creative thinking. There is evidence that exposure to or experiencing violence enhances a person's likelihood of committing violence in the future (McFall et al., 1999; Ferrajão and Oliveira, 2016; Hunter et al., 2022). That is, victims of violence are more likely to be directly driven to generate maliciously. This, however, is a rare occurrence, and this research concentrates on the bi-directional connection mechanisms of negative-malevolent creative thinking in most general groups. In the idea stimulation stage, the critical point for the linkage of the two types of thinking is whether it is value-added to the ultimate goal of personal intention, and whether they will exploit people to further their own goals (i.e., Machiavellianism) (Bochkova and Meshkova, 2019).

During the creative phase, analytical thinking predominates because negative emotions impair the individual's reassessment skills, resulting in less use of problem-oriented thinking (Perchtold-Stefan et al., 2021). When the creation is complete, the individual will again value the created product. At this stage, the individual considers the possible risks and the threats they pose to their own interests, weighs the risks against the benefits, and ponders whether to harm others in order to satisfy inner desires. Due to risk aversion and a low level of inner desire to harm others, creative thinking results that are originally malicious will be impaired in the execution stage to ensure the preservation of their own interests and become negative creative results (Mumford and Hunter, 2005; Mueller et al., 2012; Blank, 2013; Murray et al., 2017, 2018; Persson et al., 2018). Conversely, negative creative thinking outcomes can also be transformed into malevolent creative outcomes through value-added execution. Therefore, in the stage of practicing creative outcomes, the individual's intention, i.e., whether to undertake risk and whether to harm others; and whether to value-add execution to maximize benefits, are the key nodes in the linkage of the two types of thinking in that stage.

## Discussion

Malevolent creativity, according to Hao et al. (2016), maybe a component of regular employment. Negative creativity can also be seen in schooling (Meshkova et al., 2021). Terrorism, crime, theft, and espionage are examples of malevolent or negative innovation in their broadest sense (Cropley et al., 2008; Gill et al., 2013). Cheating, lying, retribution, and disinformation are examples of malevolent or negative creativity on a smaller scale (Gill et al., 2013; Harris and Reiter-Palmon, 2015; Hao et al., 2016). Based on this model, we will elaborate the linkage between negative and malevolent creativity in education from both micro and macro perspectives.

### The Linkage Model of Negative Creative Thinking and Malevolent Creative Thinking From the Micro Perspective

From a micro perspective, we consider how a course can be designed to detect and intervene with the production of negative and malevolent creative thinking in time. In the context of educational learning, the creative thinking training process is divided into three stages: idea stimulation, analysis and creation, and presentation and promotion (Zehui et al., 2019). The starting point for negative creative thinking is in the creative stimulation stage. Teachers create specific situations for students based on projects to elicit questions for inquiry; assist students in decomposing problems and goals, transforming structures and contexts, working to find multiple solutions, experimenting with multiple solutions, reverse thinking, and negotiating constructs; and dispersing and activating ideas through questioning, exploring, imagining, and expanding (Zehui et al., 2019). At this stage, teachers should pay more attention to individual creativity while focusing on group creativity, and promptly identify individual negative emotions as well as the generation of malicious motives. It has been demonstrated that expertise is a crucial driver of idea production and refinement (Hunter et al., 2022), and that people with great cognitive abilities may explore and improve ideas more successfully than those who do not have such abilities (Jaarsveld et al., 2015). Individuals create negative feelings when their own abilities do not drive the development and implementation of creative ideas, which may lead to the production of negative creative thinking (Perchtold-Stefan et al., 2021). Especially in the process of thought dispersion and decision aggregation, and group negotiation construction, Hunter et al. (2022) suggested that group influence may also be a factor in the generation of negative, malevolent creative thinking in individuals. Therefore, teachers should pay attention to individual student's emotional state and potential value orientation, and making positive guidance and care can curb negative and malevolent creative motivation in the cradle.

In the analytic creation stage, negative creative thinking and malevolent creative thinking are iteratively intertwined. The main points of this stage of thinking are design and utility analysis, which involve the integrated use of design and analytical thinking, learning from experience, integration of existing knowledge, skills, and resources, and estimation of the value of creative products. People that are creative are more prone to be dishonest (De Dreu and Nijstad, 2008; Beaussart et al., 2010; Gino and Ariely, 2012). When considering personal goals, creativity can also be dubious (Mueller et al., 2012; Gutworth et al., 2016). When an individual's creative capacity and desire to react cruelly are controlled for, positive personality traits are favorably associated with creativity (Xu et al., 2021, 2022; Wang et al., 2022). At this point, we must concentrate on guiding students' ideology and morality (Hansika, 2015; Jonason et al., 2015; Meshkova et al., 2021; Kapoor and Kaufman, 2022) in order to avoid the formation of Machiavellianism (Bochkova and Meshkova, 2019). Teachers need to provide students with relevant reference cases and learning materials according to the specific situation of the task and guide them to improve their creativity in the previous stage. Interfering with and curbing the production of negative or malevolent creative thinking in the creative stimulation stage does not mean that negative or malevolent creativity will not be produced in the analytical creation stage. Negative and malevolent creative thinking at this stage sprouts between balance and trade-offs between originality and practicality of the product orientation. Negative creative thinking (e.g., imitation, plagiarism) and malevolent creative thinking (e.g., unscrupulous destruction of others' creative results) can still occur in order to be different, and negative or malevolent creative thinking can also occur in order to highlight the value of creative products. The creation stage is an important part of the initial determination and linkage between the preliminary creative ideas and the later creative results presentation and promotion, and it is also the key to the linkage between negative and malevolent creative thinking. Therefore, multiple levels of thinking training can be done at this stage in the implementation of creative ideas and iterative adjustment of creative solutions, so as to detect students' negative and malevolent motives at different levels. Furthermore, those with higher levels of malevolent or negative creativity are less self-aware (Kapoor and Khan, 2019). As a result, teachers need to pay special attention to less self-aware kids.

Display and promotion stage teachers organize appropriate communication or display activities, and students present and share their creative results. During the display process, students and teachers will make value judgments on other works, which is one of the assessment indexes for judging the creative teaching results. Prejudice, discrimination, and anger all stimulate the production of negative, malevolent creative thinking (Kirkpatrick, 1993; Cropley et al., 2014). At this point, when the individual predicted value is not equal to the value judged by others, a negative reaction will occur. In most current creator or STEM education, the aspect of presentation and promotion is often neglected or partially missing. Many maker education stops at making a good product or simply displaying it without considering the psychological condition of the individual student, which is the key to the creation of negative and malevolent creative thinking.

### A Linkage Model of Negative and Malevolent Creative Thinking in a Macro Perspective

To look beyond the micro-framework of curriculum design, the discovery and intervention for the linkage of negative and malevolent creative thinking in the temporal dimension of individual development is not disconnected but should be a continuous spectrum with different emphases. In the primary education stage, provide students with as many opportunities as possible to experiment, to go through a process of accumulating experience, creating products, trial, and error, and gradually approaching success, and from which they learn to negotiate, cooperate, and develop non-intellectual qualities such as integrity, responsibility, strength, and fear of setbacks (Zehui et al., 2019). Students are given sufficient self-regulation to regulate their own negative emotions or motivations that may arise during the creative process. With the growth of age and school level, exposing students to a more social environment and considering more the social significance of the creative outcome in the creative process is conducive to dovetailing with innovation education at the higher education level and is conducive to helping students shape correct to moral values and better help them recognize the negative and malicious creation in the creative process.

Based on the consideration of the environment, it is particularly important to build an educational ecosystem intervention means of negative—malevolent creative thinking linkage. Creativity education is not only limited to the classroom, but through the linkage of many parties, such as school and society, it helps to collaboratively cultivate students' creativity, as well as to discover students' negative creative thinking and malevolent creative thinking in different contexts, and fully support students' positive creativity and transformation of results, effectively contributing to the creativity education.

## Conclusion

Negative creative thinking and malevolent creative thinking are different in their core categories. The two types of thinking have something in common in terms of value orientation, environmental stimulation, and subjective motivation; however, they differ in terms of value goals, thinking styles, and subject size. The identification of such differences helps to think about and study the paths of the two types of thinking more comprehensively. Negative creative thinking may be transformed into malevolent creative thinking after it is generated out of the weighing of personal interests and the realization of ultimate intentions, or it may remain negative creative thinking. Malevolent creative thinking may also become negative or malevolent creative outcomes after it is stimulated, due to risk avoidance and the value choices it ultimately practices. The value-added is the stimulus for the conversion of negative creative thinking to malevolent creative thinking, and personal intention is the key stage in the linkage of negative creative thinking to malevolent creative thinking.

From a pedagogical perspective, our creative education usually emphasizes the cultivation of creative thinking (Wilson, 2005; Baer and Kaufman, 2006; Kaufman and Beghetto, 2009; Zehui et al., 2019; Dou et al., 2021) and does not extend to the avoidance of negative and malevolent creative thinking. In the context of the current creative society, the cultivation of creative talents should consider both intervention and avoidance of negative and malevolent creative thinking. The NMTIM model proposed in this paper can be used to distinguish the difference between the two types of thinking, but it is also a conceptual prototype for the linkage model of the two types of thinking. Negative creative thinking and malevolent creative thinking are iterative in the whole creation process. There is a close interplay between the three stages of inspiration, creation, and practice. Having clarified the distinctions and connections between the two types of thinking, it is necessary to make further in-depth discussions in the following directions: (1) The linkage of negative and malevolent creative thinking in education. In the process of thinking development, we should also pay attention to students' potential negative creative thinking and make timely interventions. (2) Linking negative creative thinking and malevolent creative thinking with technological support. Technology in the age of intelligence is changing rapidly, and new technology itself is a product of creativity and a great motivation to promote creative thinking education. In particular, artificial intelligence has the potential to be “disruptive” in terms of speed, breadth, and depth. The linkage of negative creative thinking and malevolent creative thinking with technological support will become one of the important directions for the next research.

## Limitation and Future Research Directions

There are certain limitations to the research effort presented in this study. First, this study addresses a wide range of individual behaviors associated with negative creative thinking and malevolent creative thought, providing a complete understanding of the topic. We acknowledge that the linking model is purposely broad to maximize the model's accessibility and usefulness. Second, the bidirectional linkage described in this research focuses on the individual's internal thinking logic and does not involve any influencing factor variables. According to Hunter et al. (2022), social and group influences may also be factors in the generation of negative, malevolent creative thinking in individuals. As a result, future research could include extrinsic influence factors. Third, after discussing individual negative-malicious creative thinking interconnections, the study might be expanded to include group negative-malicious creative thinking relationships.

## Author Contributions

XinyuD was responsible for the writing of the article. XinyanD was responsible for the creation of the model. LJ was responsible for reviewing. All authors contributed to the article and approved the submitted version.

## Conflict of Interest

The authors declare that the research was conducted in the absence of any commercial or financial relationships that could be construed as a potential conflict of interest.

## Publisher's Note

All claims expressed in this article are solely those of the authors and do not necessarily represent those of their affiliated organizations, or those of the publisher, the editors and the reviewers. Any product that may be evaluated in this article, or claim that may be made by its manufacturer, is not guaranteed or endorsed by the publisher.
